# Interventional modalities for the prevention and management of childhood myopia in frontiers in ophthalmology: PREC strategy for myopia in children

**DOI:** 10.3389/fopht.2026.1740349

**Published:** 2026-03-19

**Authors:** Dominique Bremond-Gignac, Matthieu P. Robert, Alejandra Daruich

**Affiliations:** 1 Ophthalmology Department, APHP, Paris Cité University, Hopital Universitaire Necker-Enfants Malades, Paris, France; 2 INSERM UMRS 1138, T17, Sorbonne Paris Cité University, Paris, France; 3 Ophthalmology Department, APHP, Hopital Universitaire Necker-Enfants Malades, Paris, France; 4 Borelli Centre, UMR 9010, CNRS-SSA-ENS Paris Saclay-Paris Cité University, Paris, France

**Keywords:** children, defocus contact lenses, defocus spectacle lenses, environmental factors, low-dose atropine, myopia, strategy

## Abstract

The increasing global prevalence of myopia represents a major public health concern. This perspective article aligns with the research theme of the special issue on Interventional Modalities for the Prevention and Management of Childhood Myopia. In progressive myopia, the goal is to implement reliable early-life strategies to control myopia and reduce the risk of high myopia. We propose the PREC framework (Prevent, Recognize, Evaluate, and Control) for childhood myopia. Each component is detailed to support individualized myopia management. However, further evidence is required to establish robust guidelines and optimize management strategies, particularly for non-isolated myopia.

## Introduction

In the human evolution, nearsighted has appeared as an evolution for new activities as hunting and picking were not anymore, the daily priority. However, even if myopia has appeared as an unavoidable visual evolution, it seems that increased axial length has been over targeted creating myopia complications according to myopia level. These complications forced us to consider myopia as a disease and not only a refractive anomaly. It is important to distinguish high myopia, defined as a spherical equivalent greater than –6.00 diopters or an axial length equal to or greater than 26 mm, from pathologic myopia, in which high myopia is associated with structural changes and visual impairment. Although it remains unclear whether these two entities represent different stages of the same disease or distinct conditions, it is essential to adapt reliable strategies for myopia control since childhood to avoid at least high myopia. Every diopter spared (or gain of diopter) is an advance for children (1).

The increasing worldwide prevalence of myopia is a major public health concern, with projections suggesting that nearly 50% of the world’s population will be myopic by 2050 (2). This trend is driven by lifestyle and environmental factors—including increased screen time, prolonged near work, and reduced outdoor exposure—and shows marked geographic and ethnic variability. Genetic predisposition and gene–environment interactions play a crucial role, as children of myopic parents have a substantially higher risk. Advances in genetic and molecular research have identified numerous loci, genes, and pathways involved in ocular growth, neurotransmission, extracellular matrix remodeling, and epigenetic regulation (3). Dopamine, a key retinal neurotransmitter, plays a crucial role in regulating ocular growth, and reduced dopamine levels are associated with increased axial elongation and the development of myopia. The influence of light exposure on retinal dopamine production highlights a mechanistic link between environmental factors—particularly outdoor light exposure—and myopia progression. Transforming growth factor-β (TGF-β) plays a key role in scleral extracellular matrix remodeling, promoting scleral thinning and axial elongation of the eye—hallmark features of myopia. Increased levels of TGF-β have been reported in myopic eyes, supporting its involvement in myopia development (3).

**Figure 1:**
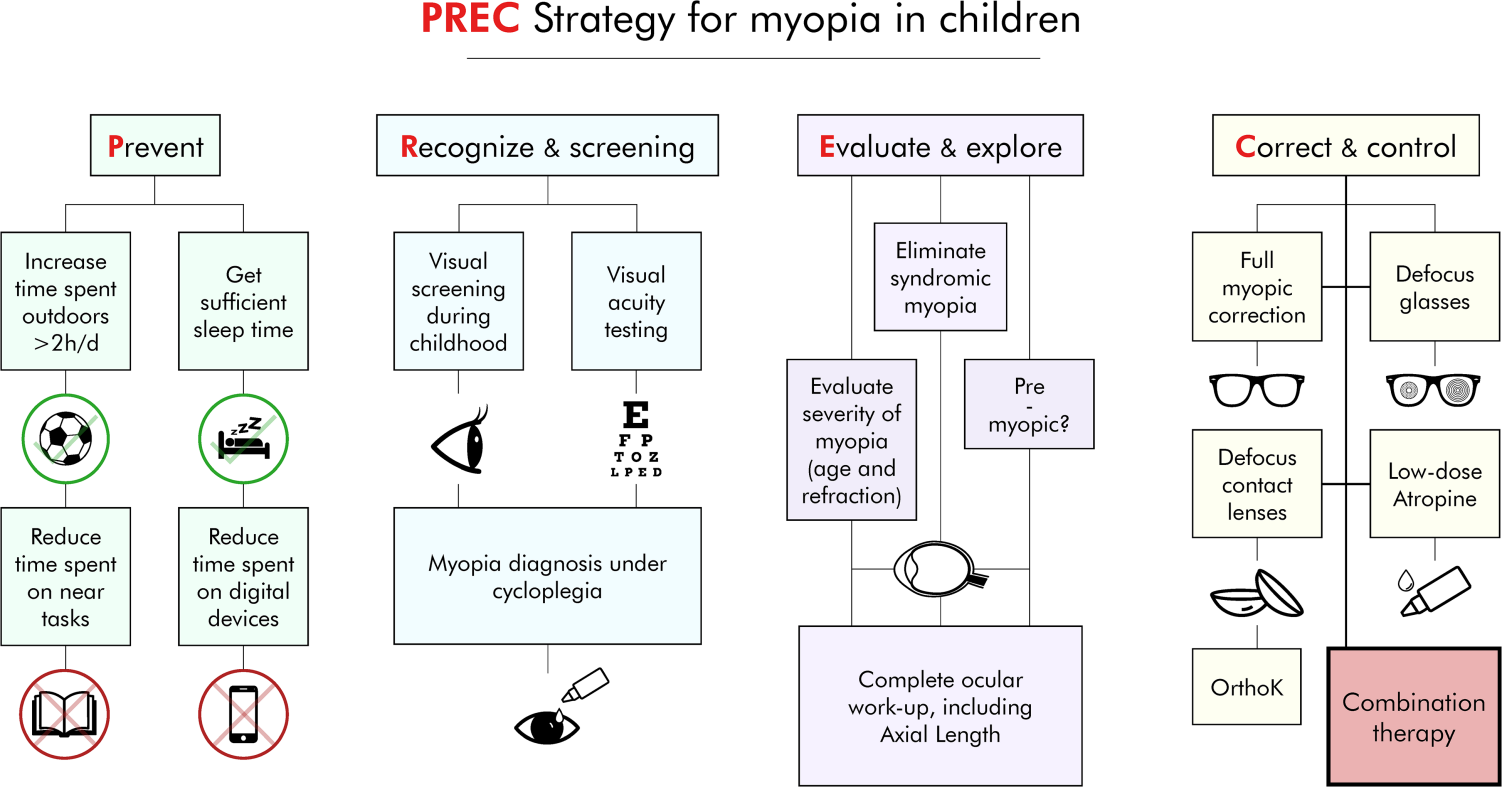
PREC strategy: Prevent, Recognize, Evaluate and control myopia in children.

PREC strategy includes: Prevent, Recognize, Evaluate and Control myopia in children (**Figure 1**) (4–8).

Prevent myopia remains essential and behavioral interventions, such as increasing time spent outdoors, reducing time devoted to near tasks—particularly those involving digital devices— and sufficient sleep time duration during school days, should be recommended to prevent the onset of myopia (9).

Recognize myopia in early childhood is the first step for myopia control. Visual screening, including visual acuity testing, photoscreening, and the cover test, allows early recognition of myopic patients.

Evaluating these children help exclude differential diagnoses and classify myopia according to its etiology. Evaluation includes mandatory cycloplegic refraction and a comprehensive ophthalmological examination—including fundus examination, measurement of intraocular pressure, and axial length. It is essential to exclude a congenital glaucoma and to differentiate isolated myopia (known as essential myopia) from non-isolated myopia, as the management approach, strategy, and follow-up can differ substantially. Non-isolated myopia includes syndromic myopia, myopia associated with retinal dystrophies, myopia associated with retinopathy of prematurity, and secondary myopias. It is important to emphasize that myopia is not progressive in many of the last situations. Moreover, the underlying mechanisms may differ substantially from those of isolated myopia, and currently available myopia control strategies have not been adequately evaluated in these specific subgroups. As a result, extrapolating data from studies on isolated, school-age myopia may be inappropriate, highlighting the need for tailored clinical management and further research to better understand disease mechanisms, natural history, and treatment responses in non-isolated myopic eyes.

Myopia control includes several strategies that have been explored mainly in isolated progressive myopia, primarily in children aged six years and older. Progressive myopia is defined as an increase in myopic refraction of more than –0.50 diopter per year or an axial length elongation exceeding 0.2 mm per year. This definition should be interpreted in the context of age-appropriate normative values for axial length growth in children, allowing excessive ocular growth to be identified early and supporting timely decisions regarding myopia prevention and control interventions (10).

Myopia control in children should remain a personalized strategy (**Table 1**). Myopia control strategies should be discussed with the family to determine the most appropriate approach for each patient and to promote adherence to both treatment and follow-up. Age and initial refractive error are key factors in selecting a control strategy, together with the familial and socioeconomic context. For instance, combined treatment approaches may be preferred in younger patients with higher degrees of myopia whereas orthokeratology or contact lenses are generally avoided in very young children. Additionally, identifying children at high risk of developing myopia—particularly those with parental myopia or lower levels of hyperopic reserve—can help target early interventions. Follow-up visits every six months should include assessment of visual acuity, refraction, and axial length.

**Table 1:** Key trials and meta-analyses on myopia onset prevention and control.

Intervention	Goal	Results	Recommandation/Reference	Clinical evidence
Outdoor time program at school	Prevention (incident myopia)	3-year myopia incidence 30.4%(intervention) vs 39.5% (control); absolute difference −9.1%	Large cluster RCT, strong pragmatic evidence for behavioral prevention He M et al. *JAMA*. 2015	I
Outdoor time with objective monitoring	Prevention	Demonstrated benefit using objective measures of outdoor exposure/light intensity	Cluster RCT, supports implementation and measurement approach He X et al. *Ophthalmology*.2022	I
Digital screen time	Prevention (risk factor)	Demonstrated risk dose-response association of time spent on digital screens Systematic review/meta-analysis of 45 studies, 335,524 participants: per +1 h/day screen time, myopia OR 1.21	Meta-analysis Observational dose-response supports counseling and policy approaches Ha A, et al. JAMA Netw Open. 2025	II
Low-dose atropine 0.05% (vs placebo) LAMP2	Prevention (incident myopia in premyopes)	2-year myopia incidence 28.4%(0.05%) vs 53.0%(placebo)	Randomized clinical trial directly tests delay of onset Yam J et al. *JAMA.* 2023	I
Atropine dose-finding network meta-analysis (8 concentrations)	Progression (and comparative dosing)	Network meta-analysis of 16 RCTs, 3272 children: 0.05% among most effective across refractive/axial outcomes with dose-dependent adverse effects	NMA informs dose selection when choosing atropine Ha A et al. *Ophthalmology.* 2022	I
Atropine 0.05/0.025/0.01% — LAMP 1	Progression	Randomized, double-blind, placebo-controlled comparison of three low concentrations; 0.05% most effective among tested doses	Core RCT establishing relative low-dose efficacy Yam J et al. *Ophthalmology.* 2019	I
Atropine long-term strategy — LAMP 3-year (continued vs washout)	Progression; stopping/”rebound”	Continued therapy superior to washout	RCT extension informs duration, discontinuation, rebound management Yam J et al. *Ophthalmology.* 2022	I
Atropine long-term strategy — LAMP 5-year	Progression; long-term outcomes	5-year follow-up supports ongoing effectiveness; retreatment if progression recurs after stopping	Long-term clinical trial evidence for maintenance/step-down plans Zhang XJ et al. *Ophthalmology*. 2024	I
Atropine 0.01% meta-analysis (worldwide)	Progression	Meta-analysis of 11 trials, 2046 children: benefit vs placebo 0.16 D/year and −0.07 mm/year at 12 months	Quantifies modest but reproducible effect size for 0.01% Navarra P et al. *Front Pharmacol.* 2025	I
Multifocal soft contact lenses	Progression	3-yearprogression: SER −0.60 D (high add) vs −1.05 D (single vision); axial growth 0.42 mm vs 0.66 mm	Randomized clinical trial pragmatic for real-world CL prescribing Walline J J et al. *JAMA*.2020	I
Multifocal soft contact lenses	Progression	Structured evidence synthesis (12 studies; 11 Level I) supports efficacy/safety of multifocal soft contact lenses	“Guideline-like” AAO report for clinical adoption and safety considerations Cavuoto AM et al. *Ophthalmology.* 2025	I
Orthokeratology	Progression	NMA of 30 RCTs, 5422 eyes: orthokeratology reduced axial elongation by −0.15 mm/year vs control	NMA, comparative efficacy across modalities; positions Ortho-K vs alternatives Huang J, et al. *Ophthalmology*. 2016.	I
Combination therapy (Ortho-K + atropine 0.01%) —	Progression (rapid progressors)	NMA of 80 RCTs, 27,103 eyes: combined treatments among most effective with AXL −0.47 mm/year and SER + 0.81 D/year vs control Supports step-up to combination in high-risk/fast progression settings	NMA, supports step-up to combination in high-risk/fast progression settings Zhang G, et al. *Eye* (Lond). 2023.	I
Myopia-control spectacle lenses (AMDT)	Progression	RCT: 144 randomized (140completed); difference vs single vision 0.39 D and 0.17 mm at 12 months	Double-masked RCT supports newer lens designs with objective endpoints Wang M et al. *Ophthalmology*. 2025	I
Myopia-control spectacle lenses —	Progression	Meta-analysis of 23 RCTs, 13,315 subjects: reduced axial elongation −0.15 mm and SER −0.31 D vs single vision; HAL subgroup AXL −0.28 mm, SER −0.52 D	Meta-analysis, RCT-only synthesis to rank spectacle lens designs (HAL/DIMS-type) D’Andrea L et al. *Ophthalmology*. 2026	I

Peripheral refraction of the retina can influence ocular growth. Spectacle lenses, including Defocus-Incorporated Multiple Segments (D.I.M.S.) and Highly Aspherical Lenslet (H.A.L.) designs, which induce myopic peripheral defocus, have been shown to reduce myopia progression and axial elongation after more than 6 and 5 years of follow-up, respectively (11–13). Defocus contact lenses, such as MiSight, and orthokeratology using specially designed reverse-geometry rigid gas-permeable lenses have also been shown to reduce myopia progression after 6 and 2 years of use, respectively (14, 15). Orthokeratology uses customized rigid contact lenses worn overnight to temporarily modify corneal curvature, reducing central corneal power while increasing curvature in the mid-peripheral cornea. Atropine eye drops have shown dose-dependent efficacy in myopia control, with higher concentrations (0.1% to 1%) providing greater effect but also associated with a more pronounced rebound phenomenon after treatment cessation (16, 17). The exact mechanism by which atropine slows myopia progression remains unclear and is now thought to extend beyond its cycloplegic, anti-accommodative effects. Current evidence suggests atropine modulates ocular growth through non-accommodative pathways, including muscarinic receptor inhibition, reduced scleral remodeling, and decreased cellular proliferation. Additional proposed mechanisms include enhanced retinal dopamine release and altered growth factor signaling, particularly involving TGF-β and other retinal and choroidal mediators (3). Finally, combined treatment strategies appear to be more effective in controlling myopia progression than monotherapy (18). Dopamine agonists and other innovative drugs are under development and may lead to prevent and control myopia in children.

Overall, with the global prevalence of myopia steadily increasing, current evidence supports the implementation of strategies to prevent and control myopia. The PREC strategy aims to prevent, recognize, evaluate and control progressive isolated myopia in children through a personalized approach, tailored to each child’s family, lifestyle and socioeconomic context. However, further evidence is needed to establish guidelines and optimal management strategies for non-isolated myopia.

## Data Availability

The raw data supporting the conclusions of this article will be made available by the authors, without undue reservation.
