# Supplementary material for: Interventional modalities for the prevention and management of childhood myopia in frontiers in ophthalmology: PREC strategy for myopia in children
Source: Front Ophthalmol (Lausanne). 2026 Mar 19;6:1740349. doi: 10.3389/fopht.2026.1740349 (PMC13043351; doi:10.3389/fopht.2026.1740349)
Supplement: Supplementary file 1 [file DataSheet1.pdf]

Refractive myopia	Lens ectopia	
Axial myopia	Congenital syndromic myopia	Collagenopathies (Stickler syndromes, Knobloch syndrome, etc.), proteoglycanopathies (Wagner syndrome), lamininopathies
	Infantile / childhood myopia	Congenital/infantile glaucoma
		Retinopathy of prematurity
		Inherited retinal disorders
		Fibrillinopathies (Marfan syndrome, Ehlers Danlos syndrome, etc.)
		Panocular diseases (Aniridia, Albinism, etc.)

Table 1. Classification of non-isolated myopia
